# MRI of Wallerian Degeneration in the Brainstem: A Pictorial Essay

**DOI:** 10.5334/jbsr.2585

**Published:** 2021-10-14

**Authors:** Nico Hustings, Marc Lemmerling

**Affiliations:** 1 UZ Leuven, BE; 2 AZ Sint-Lucas, BE

**Keywords:** MRI, Wallerian degeneration, cerebral injury, corticospinal tract, peduncular atrophy, prognosis

## Abstract

Wallerian degeneration of the cerebral peduncle is a common MRI finding after cerebral injury. The degree of peduncular atrophy reflects the extent of damage in the corticospinal tract. The acute phase of Wallerian degeneration is visible with Diffusion-Weighted-Imaging. New investigation with Diffusion-Tensor-Imaging quantifies Wallerian degeneration in the subacute ischemic phase and is a good predictor for later functional recovery after stroke.

## Introduction

Wallerian degeneration (WD) in the central nervous system produces a contiguous tract of gliosis, starting at a damaged region of cerebral cortex and running through the deeper brain structures according to the topography of the involved white-matter tracts. The process starts immediately after injury but evolves to complete after months-to-years. On Magnetic Resonance Imaging (MRI), chronic WD of the corticospinal tract can be observed as shrinkage of the ipsilateral cerebral peduncle [1]. We herein illustrated WD via selection of a range of MRI’s with atrophied cerebral peduncles secondary to various causes.

## Ischemic stroke

**Figure 1:**
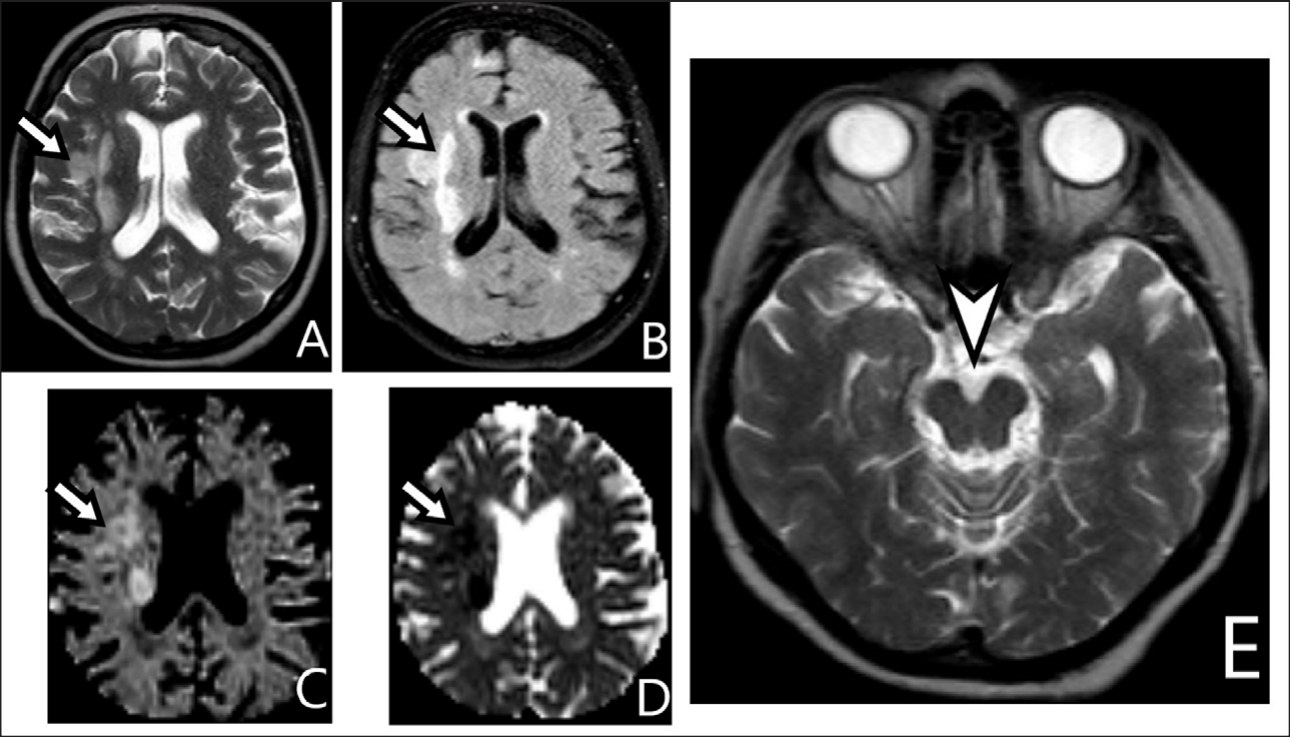
The perfusion area of the right middle cerebral artery is hyperintense on axial T2-weighted Imaging (WI) **(A)** and Fluid-Attenuated Inversion-Recovery (FLAIR) **(B)**, also hyperintense on Diffusion-Weighted Imaging (DWI) **(C)** and hypointense on Apparent-Diffusion Coefficient (ADC) map **(D)** (*arrows*). The cerebral peduncles are symmetric on T2-WI **(E)**, (*arrowhead*).

**Figure 2:**
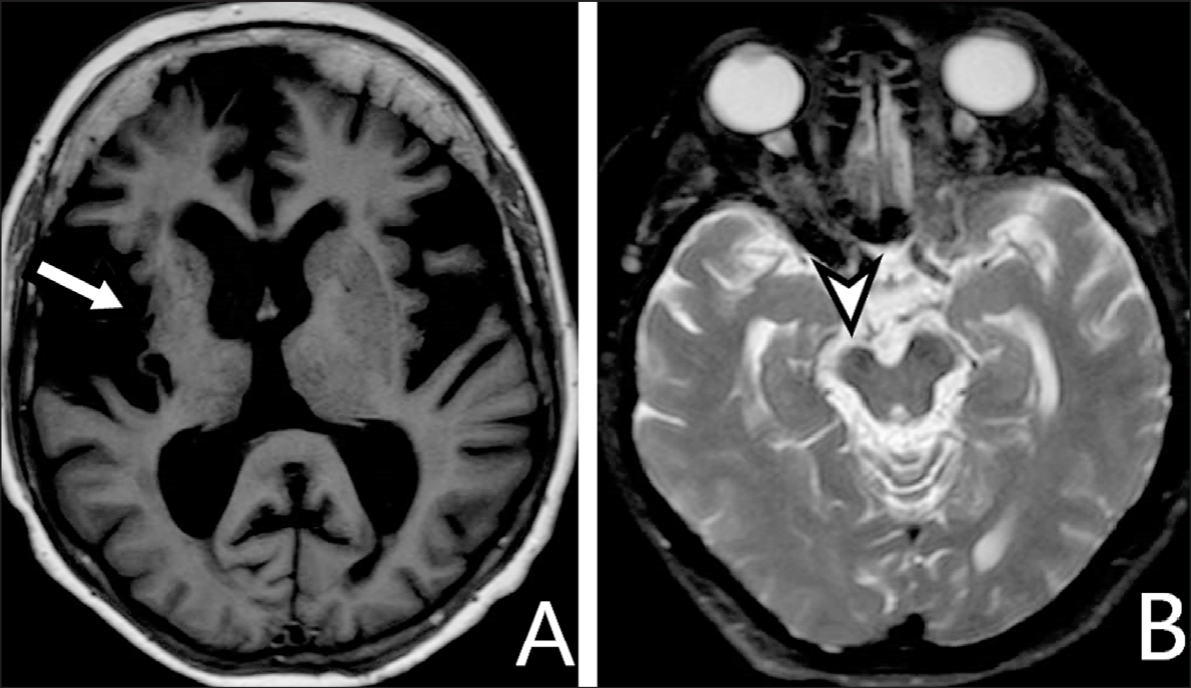
**A:** *axial T1-WI*, **B:** *T2-WI*. Tissue loss in the right insula and basal ganglia (*arrow*). The ipsilateral cerebral peduncle is atrophied by WD (*arrowhead*).

MRI of a 65-year-old woman with left hemiplegia caused by an embolism. MRI during acute stroke (***Figure 1***) and three years later (***Figure 2***).

## Hemorrhagic stroke

**Figure 3:**
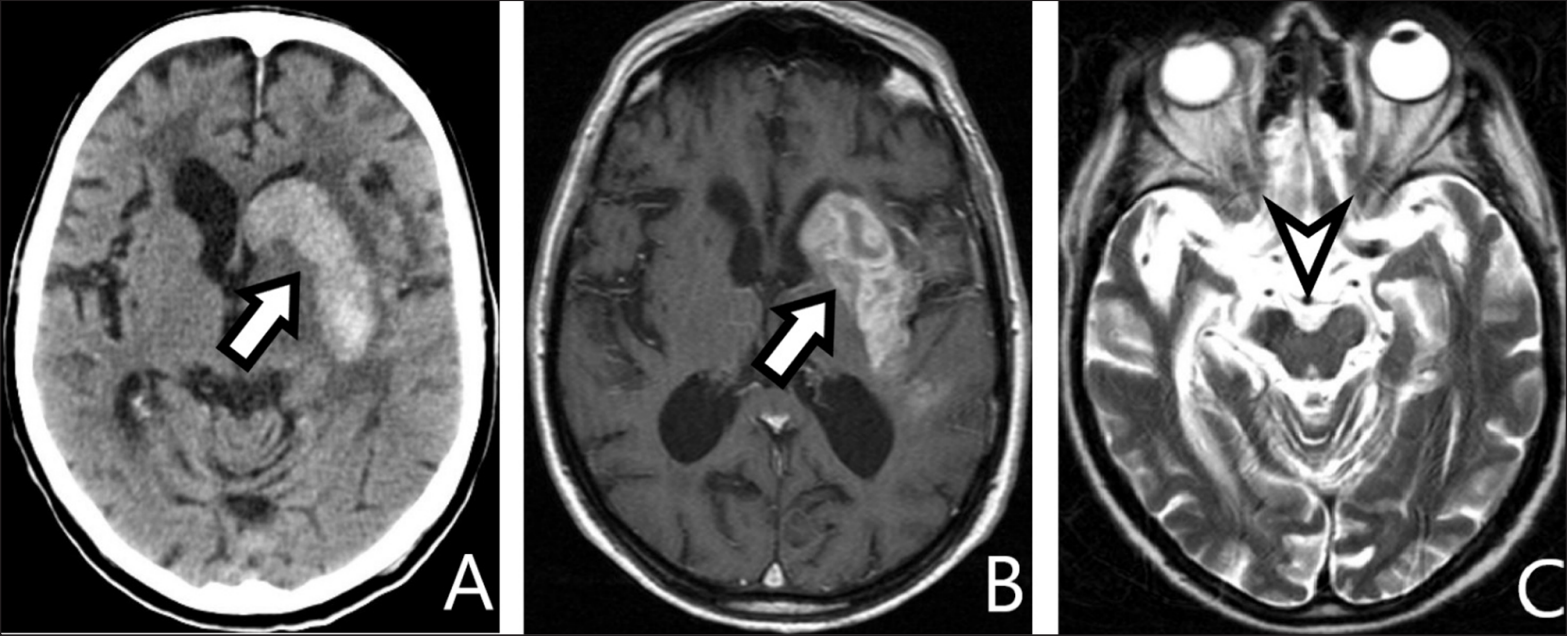
**A:** *axial soft-tissue window CT*, **B:** *T1-Gd*, **C:** *T2-WI*. Left intracerebral hemorrhage in the caudate and lentiform nuclei, hyperdense on CT and hyperintense on MRI (*arrows*). The cerebral peduncles symmetric (*arrowhead*).

**Figure 4:**
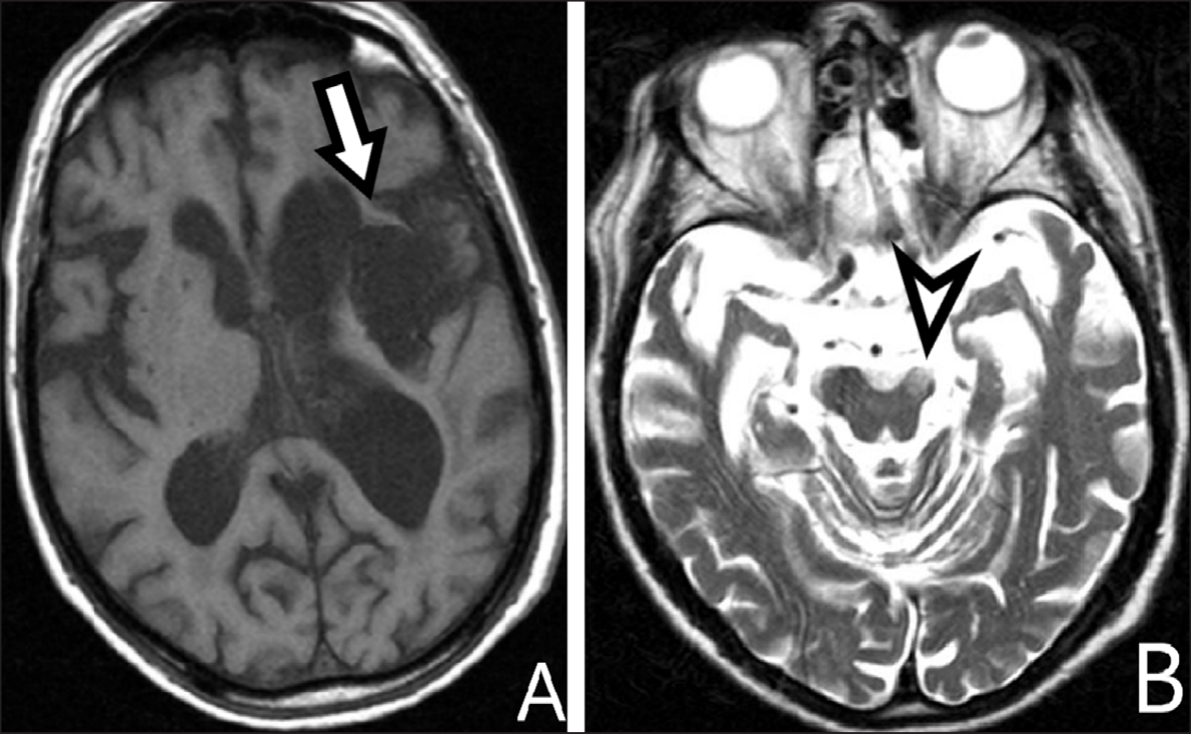
**A:** *axial T1-WI*, **B:** *T2-WI*. Atrophy of the left basal ganglia shown by a hypointense zone on T1-WI (*arrow*), accompanied by atrophy of the left mesencephalon (*arrowhead*).

MRI of an 83-year-old man with aphasia and right hemiplegia caused by intracerebral hemorrhage. Compare computed tomography (CT) and MRI during the hemorrhage (***Figure 3***) are compared to MRI three years later (***Figure 4***).

## Brain tumor

**Figure 5:**
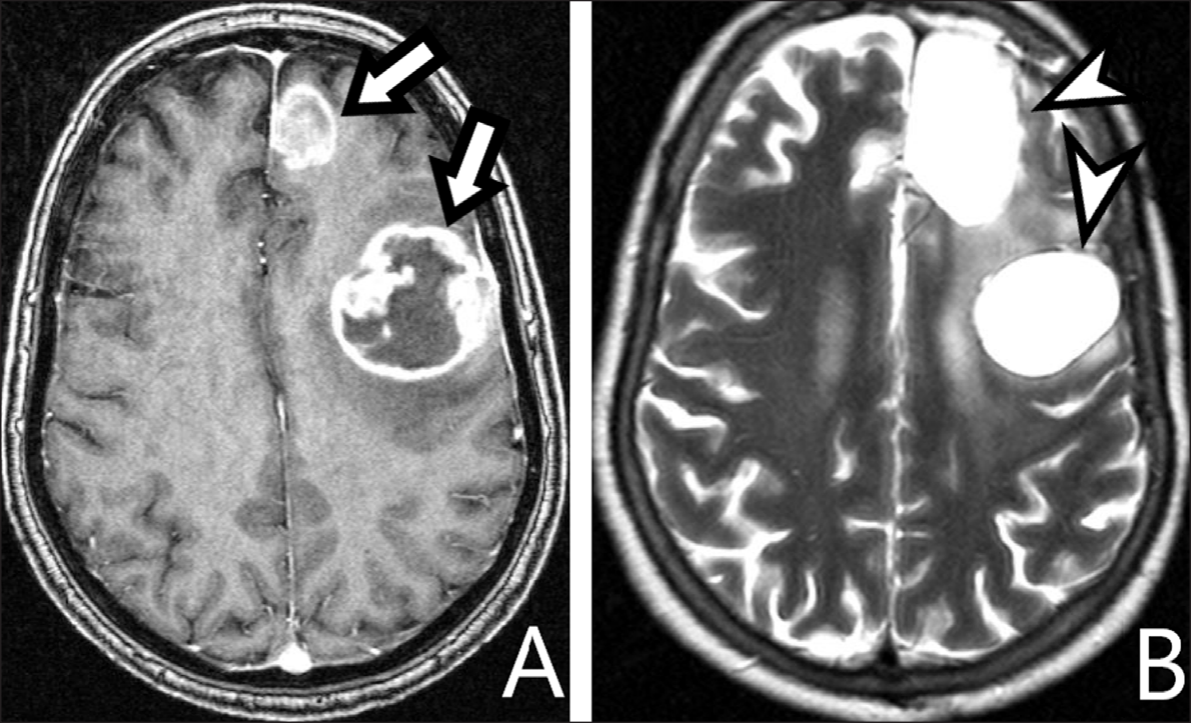
**A:** *axial preoperative T1-Gd*, **B:** *postoperative T2-WI*. Two heterogeneous contrast-enhancing left frontal tumors (*arrows*). Zones of tissue loss, filled with cerebrospinal fluid (*arrowheads*).

**Figure 6:**
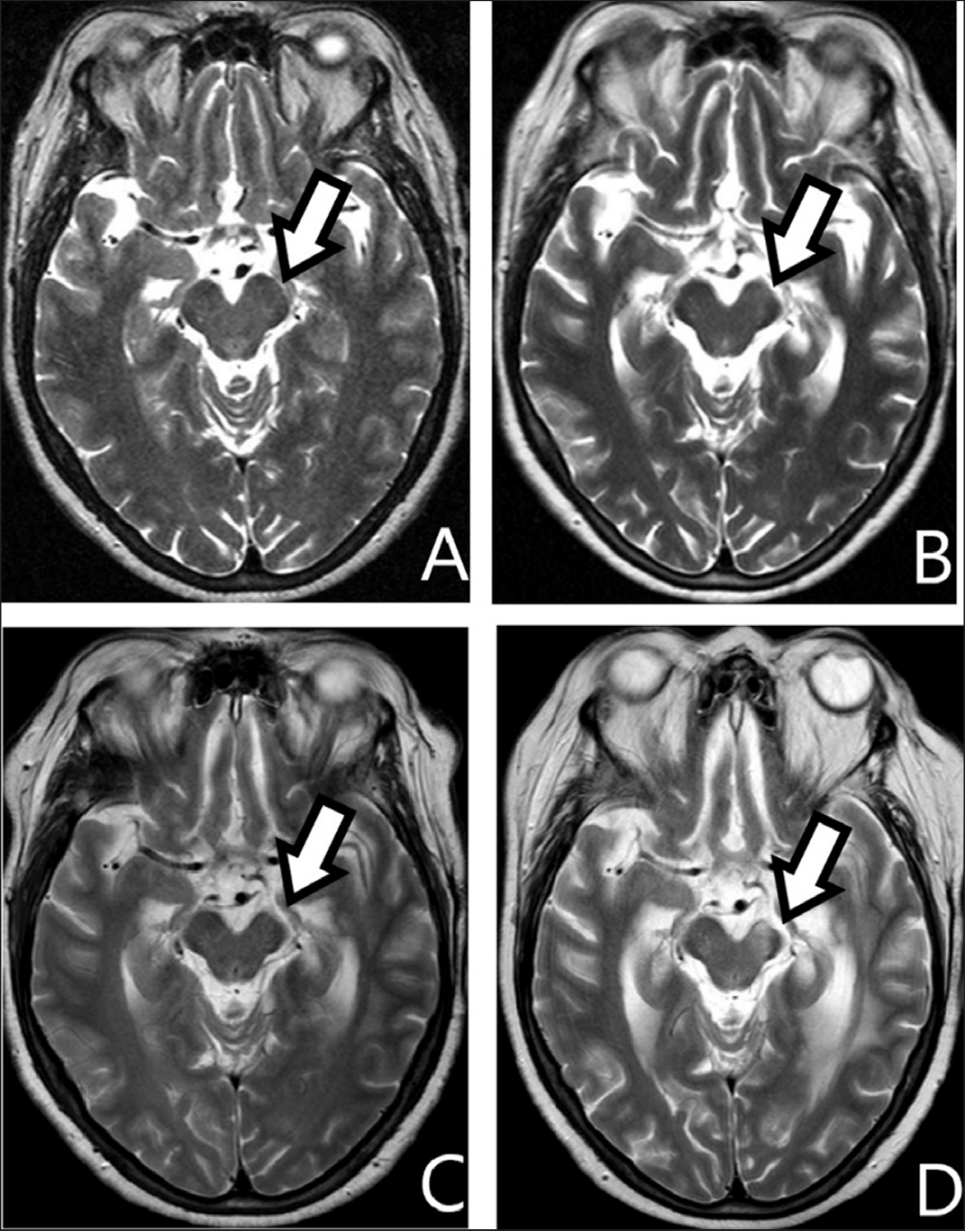
**A, B, C** *and* **D:** *axial T2-WI; 0, 8, 16 and 34 months postoperative, respectively*. With increasing time after brain surgery, notice the progression of WD in the ipsilateral mesencephalon *(arrows)*.

MRI of a 61-year-old woman with glioblastoma multiforme in the left cerebrum. ***Figure 5*** shows the lesions on preoperative and postoperative MRI’s and ***Figure 6*** displays the evolution of WD with prolonged time after surgery (***Figure 6***).

## Traumatic brain injury

**Figure 7:**
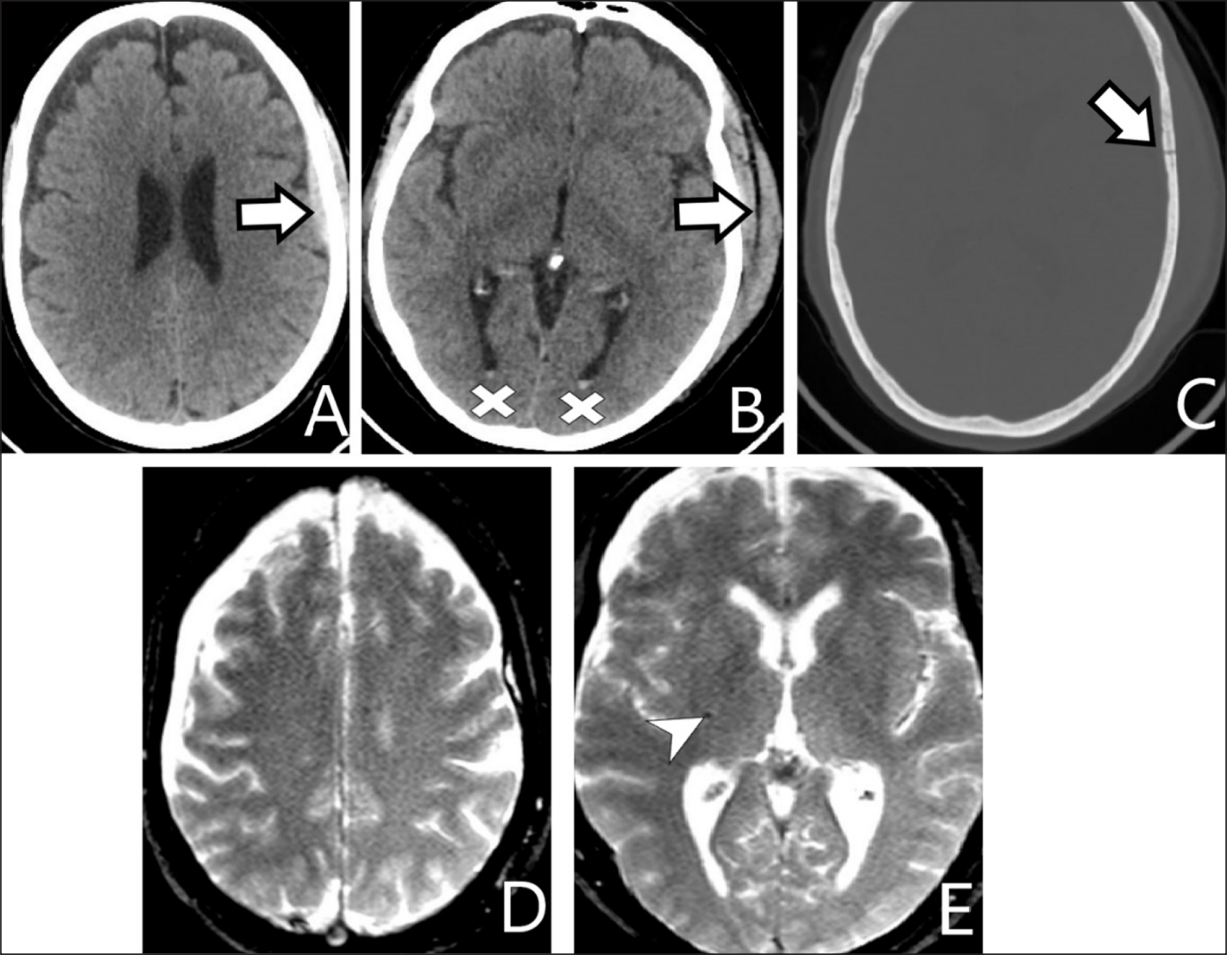
**A** *and* **B:** *axial soft-tissue window CT*, **C:** *bone window CT*, **D** *and* **E:** T2*-WI. Left frontal epidural hematoma and subgaleal hemorrhage with an underlying skull fracture (*arrows*). Small intraventricular bleedings in de occipital horns (*cross*). Diffuse petechial bleeds, one particular focal hemorrhage in the posterior limb of the right internal capsule (*arrowhead*).

**Figure 8:**
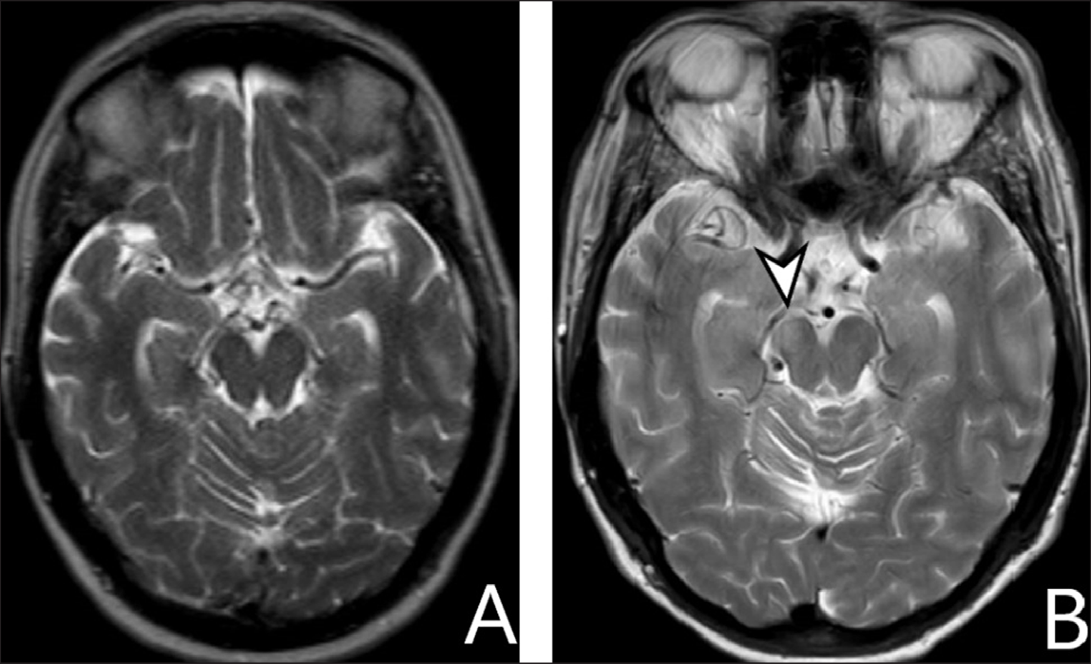
**A** *and* **B:** *axial T2-WI; 0 and 12 months after trauma, respectively*. Symmetrical cerebral peduncles at start. WD in the right cerebral peduncle one year after trauma (*arrowhead*), this atrophy correlates with the petechial bleeding in the posterior limb of the internal capsule on Figure 7.

MRI of a 53-year-old woman with brain trauma due to a fall. Eventually she developed spastic paresis, especially on the left side. ***Figure 7*** shown the CT and MRI at time of trauma, and ***Figure 8*** shows the respective size and T2-WI signal intensity of the cerebral peduncles over time (***Figure 8***).

## Large arachnoid cyst

**Figure 9:**
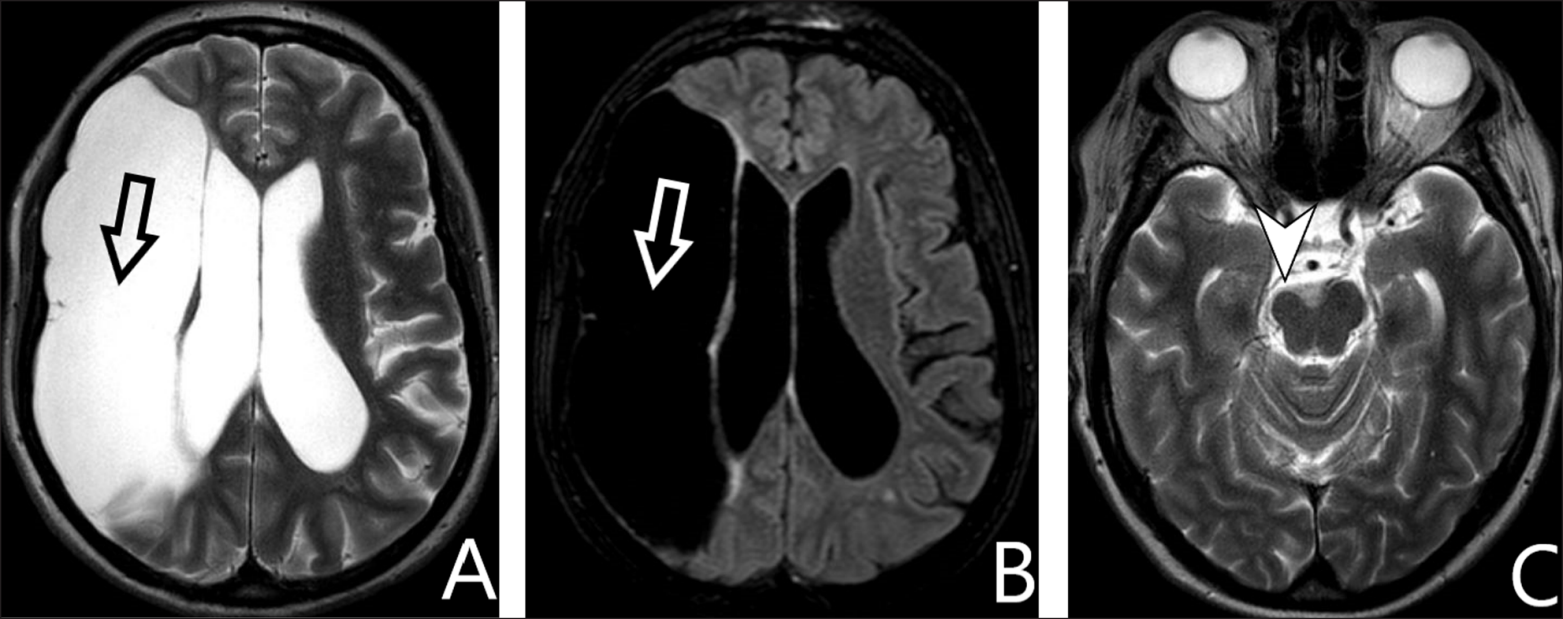
**A:** *axial T2-WI*, **B:** *FLAIR*, **C:** *T2-WI*. Right frontoparietal large arachnoid cyst, hyperintense on T2-WI and hypointense on FLAIR (*arrows*). Atrophy of the right cerebral peduncle (*arrowhead*).

MRI of a 42-year-old woman with a large arachnoid cyst (***Figure 9***).

## Cerebral cortical hypoplasia

**Figure 10:**
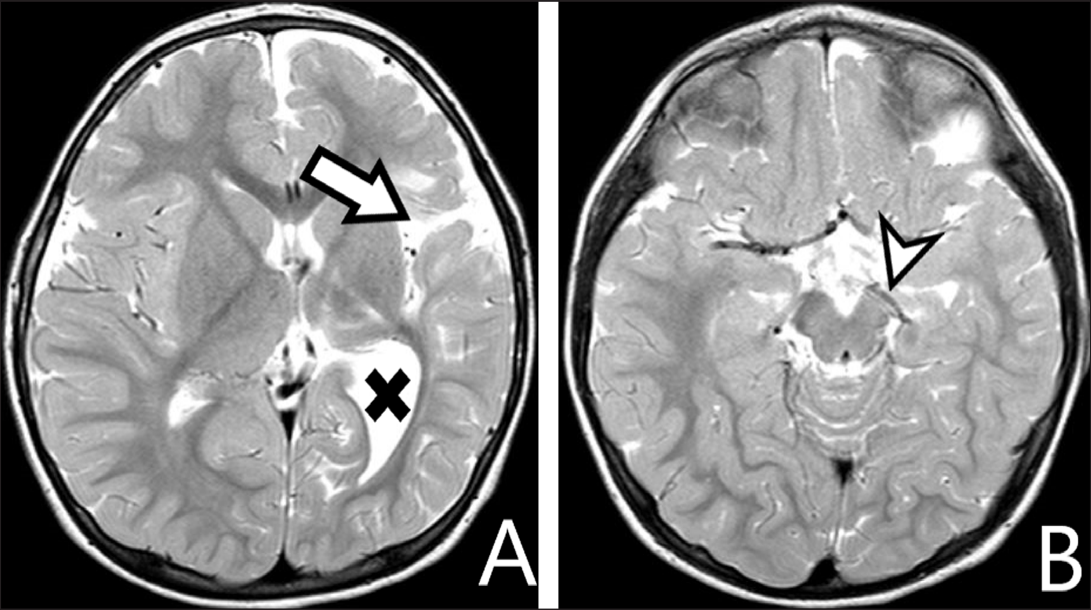
**A** *and* **B:** *axial T2-WI*. Underdeveloped left cerebrum (*arrow*), with secondary widening of the left lateral ventricle (*cross*). Left-sided mesencephalic atrophy by WD (*arrowhead*).

MRI of a 2-year-old girl with hypoplastic left cerebrum (***Figure 10***).

## Relevance of Wallerian degeneration on MRI

The degree of poststroke and posthemorragic peduncular atrophy is correlated with size of cerebral injury [2,3]. Deduced from our cases, it seems likely that this also applies for other mechanisms of injury.

In addition to the chronic atrophy of the cerebral peduncle on the long term, the acute signs of WD are also visible on MRI by Diffusion-Weighted-Imaging. It is important to recognize these diffusion-restricted areas as acute WD from damage higher in the ipsilateral corticospinal tract, and not to mistake these with secondary zones of infarction [4].

More recent investigation with Diffusion-Tensor-Imaging brought more insight on the progress of WD by the measurements of fractional anisotropy: the process of WD in the subacute phase of ischemia proved to be a predictor for later functional recovery [5].

## Conclusion

Cerebral injury frequently leads to Wallerian degeneration of the cerebral peduncle. It reflects degree of cerebral damage and predicts functional recovery, making it worth mentioning in the radiologic report.
